# NOX1 Inhibition Attenuates Kidney Ischemia-Reperfusion Injury via Inhibition of ROS-Mediated ERK Signaling

**DOI:** 10.3390/ijms21186911

**Published:** 2020-09-21

**Authors:** Hee-Yeon Jung, Se-Hyun Oh, Ji-Sun Ahn, Eun-Joo Oh, You-Jin Kim, Chan-Duck Kim, Sun-Hee Park, Yong-Lim Kim, Jang-Hee Cho

**Affiliations:** Division of Nephrology, Department of Internal Medicine, School of Medicine, Kyungpook National University, Kyungpook National University Hospital, Daegu 41944, Korea; hy-jung@knu.ac.kr (H.-Y.J.); ttily@nate.com (S.-H.O.); ggumsuni@hanmail.net (J.-S.A.); oej1124@naver.com (E.-J.O.); pinkqic1004@naver.com (Y.-J.K.); drcdkim@knu.ac.kr (C.-D.K.); sh-park@knu.ac.kr (S.-H.P.); ylkim@knu.ac.kr (Y.-L.K.)

**Keywords:** NOX1, ML171, reactive oxygen species, ERK, ischemia-reperfusion injury, acute kidney injury

## Abstract

The protective effects of nicotinamide adenine dinucleotide phosphate (NADPH) oxidase (NOX) 1 inhibition against kidney ischemia-reperfusion injury (IRI) remain uncertain. The bilateral kidney pedicles of C57BL/6 mice were clamped for 30 min to induce IRI. Madin–Darby Canine Kidney (MDCK) cells were incubated with H_2_O_2_ (1.4 mM) for 1 h to induce oxidative stress. ML171, a selective NOX1 inhibitor, and siRNA against NOX1 were treated to inhibit NOX1. NOX expression, oxidative stress, apoptosis assay, and mitogen-activated protein kinase (MAPK) pathway were evaluated. The kidney function deteriorated and the production of reactive oxygen species (ROS), including intracellular H_2_O_2_ production, increased due to IRI, whereas IRI-mediated kidney dysfunction and ROS generation were significantly attenuated by ML171. H_2_O_2_ evoked the changes in oxidative stress enzymes such as SOD2 and GPX in MDCK cells, which was mitigated by ML171. Treatment with ML171 and transfection with siRNA against NOX1 decreased the upregulation of NOX1 and NOX4 induced by H_2_O_2_ in MDCK cells. ML171 decreased caspase-3 activity, the Bcl-2/Bax ratio, and TUNEL-positive tubule cells in IRI mice and H_2_O_2_-treated MDCK cells. Among the MAPK pathways, ML171 affected ERK signaling by ERK phosphorylation in kidney tissues and tubular cells. NOX1-selective inhibition attenuated kidney IRI via inhibition of ROS-mediated ERK signaling.

## 1. Introduction

Kidney ischemia/reperfusion injury (IRI), which is the interruption and restoration of blood flow, is a basic pathophysiology of acute kidney injury associated with high mortality and morbidity. IRI causes oxygen and nutrition deficiency, inflammatory cell infiltration, oxygen-derived reactive oxygen species (ROS) or nitrogen-derived reactive nitrogen species generation, microvascular damage, and ultimately tissue damage [1,2,3]. Excessive ROS is among the most important contributors of tissue damage by inducing oxidative damage of deoxyribonucleic acid, proteins, and lipids [4,5,6,7,8].

Nicotinamide adenine dinucleotide 3-phosphate (NADPH) oxidase (NOX) is a major enzyme that uses NADPH to catalyze oxygen conversion to superoxide and produce ROS. Seven NOX isoforms have been identified including NOX1–5, Duox1, and Duox2 [9]. In particular, NOX1-dependent ROS production contributes to cell signaling, cell growth, angiogenesis, motility, and blood pressure regulation [10,11,12]. Previous studies have reported that NOX1 upregulation was involved in cisplatin-induced kidney injury [13] and NOX1 inhibitor has a protective effect on lung IRI by suppressing inflammatory and autophagy activation [14]. Although several antioxidants and anti-inflammatory agents [15,16,17,18,19] have effects on ROS production and inflammatory reactions, and NOX4 inhibitor [20] has been used in experimental studies to prevent or decrease IRI-induced kidney damage, the protective effect of NOX1 inhibition against kidney IRI is not totally understood.

This study’s aim is to identify the effect of NOX1 inhibition on the recovery from IRI through ROS suppression and related mechanisms. We used a pharmacological inhibitor specific for NOX1 (2-acetylphenothiazine, ML171) [21] and siRNA against NOX1.

## 2. Material and Methods

### 2.1. Animals

Eight-week-old male C57BL/6 mice that weighed 22–25 g (Samtako, Osan, Korea) were used. They were housed with a free access to standard chow and water and were kept in a 12 h light/dark cycle. They were divided randomly into six groups as follows: control vehicle (Con + Veh, *n* = 5), control with 60 mg/kg ML171 (Con + ML171, *n* = 5), sham operation with vehicle (Sham + Veh, *n* = 6), sham operation with 60 mg/kg of ML171 (Sham + ML171, *n* = 6), ischemia-reperfusion vehicle (IRI + Veh, *n* = 8), and ischemia-reperfusion with 60 mg/kg of ML171 (IRI + ML171, *n* = 8). ML171 (MedChemExpress, Monmouth Junction, NJ 08852, USA) was dissolved in 10% DMSO, 40% PEG300, 5% Tween-80, and 45% normal saline. Animals were injected intraperitoneally with a single daily dose of ML171 (60 mg/kg) or vehicle before 24 h for bilateral IRI and were sacrificed by cardiac puncture under anesthesia at 24 h after reperfusion. Blood and kidneys were harvested for the analyses. Animal experiments were performed according to the guidelines approved by the Animal Care and Use Committee at the Kyungpook National University (KNU-2017-0013).

### 2.2. Induction of Kidney IRI

For ischemia induction, the mice were anesthetized using isoflurane inhalation and kidney pedicles were completely occluded for 30 min using a microaneurysm clamp. After 30 min of ischemia, the artery clamp was removed to allow reperfusion, and the skin was closed. Identical surgical treatment was performed on sham-operated animals except for the clamping of the kidney pedicles. During the operation, animals were maintained at a temperature of 36.5–37 °C using a temperature-controlled heating device (Harvard Bioscience, Holliston, MA, USA).

### 2.3. Kidney Function and Histopathological Studies

In mouse serum, blood urea nitrogen (BUN) and creatinine (Cr) levels were evaluated by GCLabs (Yongin, Korea) using the Cobas 8000 modular analyzer system (Roche, Germany). Kidney tissues from each experimental group were immersion-fixed with 4% paraformaldehyde (pH 7.4) and then embedded in paraffin. Two-micrometer tissue sections were prepared and stained with periodic acid-Schiff (PAS) and Masson’s trichrome using standard protocols for the determination of histological changes and collagen deposition, respectively. Immunohistochemical analysis of kidney tissues detected the Nox-1 (1:100, ab121009, Abcam) and Nox-4 proteins (1:100, MA5-32090, Invitrogen).

### 2.4. Cell Culture Treatment

Madin-Darby Canine Kidney (MDCK) cells were obtained from the American Type Culture Collection (CCL-34™, Manassas, VA, USA), which were maintained in Eagle’s Minimum Essential Medium (EMEM, ATCC^®^30-2003™) and supplemented with 10% fetal bovine serum at 37 °C in a humidified atmosphere of 5% CO_2_ and 95% air. Cultured MDCK cells were plated on 96-well plates (1.0 × 10^4^ cells/well) for intracellular ROS measurement and on 12-well plates (1.0 × 10^5^ cells/well) for real-time reverse transcriptase-polymerase chain reaction (RT-PCR) and on 6-well plates (2.0 × 10^5^ cells/well) for measurement of caspase-3 activity and immunoblot analysis. For each experiment, 80–85% confluent cells were incubated with serum-free media for 24 h and were placed into the 0.2% FBS-added medium, which were treated with (1) only medium, (2) ML171 (1 μM), (3) ML171 (2.5 μM), (4) H_2_O_2_ (1.4 mM), (5) H_2_O_2_ (1.4 mM) + ML171 (1 μM), or (6) H_2_O_2_ (1.4 mM) + ML171 (2.5 μM) for an additional 48 h and were pretreated with ML171 (1 μM or 2.5 μM) for 1 h and treated with H_2_O_2_ (1.4 mM). To further study the effect of Nox-1 inhibition, MDCK cells were transiently transfected with 100 nM siRNA against Nox-1 (AccuTarget™ SMART pool customized siRNA, Bioneer, Daejeon, Korea) or nontargeting siRNA (SignalSilence^®^ Control siRNA, #6568, Cell signaling, Danvers, MA, USA) using Lipofectamine RNAiMax (Thermo Fisher Scientific, Waltham, MA, USA) for 6 h. Subsequently, cells were incubated with H_2_O_2_ (1.4 mM) for 48 h.

### 2.5. Hydrogen Peroxide Assay

Extracellular H_2_O_2_ was measured using the Amplex Red Hydrogen Peroxide Assay Kit (Thermo Fisher Scientific) in accordance with the manufacturer’s instructions. Briefly, to detect H_2_O_2_ released from mice kidney and treated MDCK cells, lysis buffer or culture media (50 mL) were reacted with the Amplex Red reagent, along with horseradish peroxidase, to produce resorufin, a red fluorescent oxidation product. Its fluorescence was determined at 530 nm excitation and 590 nm emission using a fluorescence microplate reader (Molecular Devices, Sunnyvale, CA, USA). The concentrations of H_2_O_2_ were calculated using standard curves.

### 2.6. Intracellular ROS Measurement

Mice kidney and MDCK cells were stained using 10 µM 2′,7′-dichlorodihydrofluorescein diacetate (H_2_DCFDA; Molecular Probes, Eugene, OR, USA) for 40 min and visualized using fluorescence microscopy (Nikon, Tokyo, Japan). To quantitatively measure fluorescence signal intensity, the stained kidney tissues and cells in 96-well plates were incubated with lysis buffer (0.1% Triton X-100 plus 0.5M EDTA in PBS), and the intensity was measured at 480 nm excitation and 520 nm emission using a fluorescence microplate reader (Molecular Devices Corp., Silicon Valley, CA, USA). The value of the fluorescence signal was normalized based on the total amount of cellular protein and then expressed as a percentage of the control.

### 2.7. Measurement of Caspase-3 Activity

Caspase-3 activity in the mice kidney and MDCK cells was measured using a colorimetric assay kit (Sigma-Aldrich) in accordance with the manufacturer’s protocol. In brief, kidney homogenates were incubated with the fluorometric caspase-3 substrate, Ac-DEVD-pNA, in the assay buffer. To account for nonspecific hydrolysis of the substrate, a control reaction mixture containing the caspase-3 inhibitor, acetyl-DEVD-CHO, in the assay buffer was used. Both mixtures were incubated for 90 min at 37 °C, with the absorbance being read at 405 nm.

### 2.8. TUNEL Assay 

Apoptosis was investigated by terminal deoxynucleotidyl transferase-mediated dUTP <nick end labeling (TUNEL) assay using the In Situ Cell Death Detection Kit (Roche, Mannheim, Germany), Fluorescein, for fluorescence and the Click-iT^TM^ TUNEL colorimetric IHC Detection Kit (Life Technologies, Carlsbad, CA, USA) for immunohistochemistry. Briefly, treated cells were fixed with 4% paraformaldehyde for 1 h at room temperature and then permeabilized in the 0.1% Triton X-100/0.1% sodium citrate for 2 min at 4 °C. After washing with PBS, the cells were incubated with 50 uL TUNEL reagent mixture for 1 h at 37 °C and then counterstained with 4′,6-diamidino-2-phenylindole (DAPI; Sigma, St. Louis, MO, USA) to detect cell nucleus for 1 min. Finally, the cells were mounted with the Prolong Gold anti-fade reagent (Invitrogen, Eugene, OR, USA) and then observed under a confocal microscope (Carl Zeiss, Göttingen, Germany). The number of TUNEL-positive cells was randomly counted (three to five sections per experiment). The percentage of apoptotic cells was calculated as a percentage of the TUNEL-positive cell-to-DAPI ratio. For immunohistochemistry of TUNEL assay, after treating with terminal deoxynucleotidyl transferase (TdT) reaction buffer for 10 min at 37 °C, the TdT reaction mixture was added for 60 min at 37 °C. The streptavidin-peroxidase conjugate solution was incubated for 30 min at room temperature. Then, the sections were washed and mixed with the 3,3′-diaminobenzidine (DAB) reaction to produce a brown color and then counterstained with Mayer’s hematoxylin.

### 2.9. Quantitative RT-PCRs

Total RNA was extracted from treated MDCK cells and kidney tissues using Trizol (Invitrogen, Waltham, MA) in accordance to the manufacturer’s instructions. One microgram of total RNA was reverse transcribed to cDNA using PrimeScript cDNA Synthesis kit (TaKaRa Shuzo Co., Ltd., Otsu, Japan). Quantitative PCR was performed in the synthesized cDNA using the StepOne Plus Real-time PCR system (Applied Biosystems, Foster City, CA, USA) with SYBER Green PCR master mix (Life Technologies, Carlsbad, CA, USA). The qRT-PCRs were performed in duplicate. The transcript level of target genes was calculated using the 2^-ΔΔCT^ method. All primers used for qRT-PCR were designed using the Primer Express 3.0.1 software (Applied Biosystems, Foster City, CA, USA), which are listed in Table 1.

### 2.10. Immunoblot Analysis

Immunoblot analysis detected the marker proteins of apoptosis and mitogen-activated protein kinase (MAPK) pathway, and 20 µg of protein was separated using 10% SDS-polyacrylamide gel electrophoresis and transferred to a nitrocellulose membrane, which was blocked with 10% skimmed milk for 1 h at room temperature and incubated overnight at 4 °C with primary antibodies and then incubated with a horseradish peroxidase-conjugated secondary antibody (Dako, Glostrup, Denmark) for 1 h at room temperature and detected using advanced ECL reagents (Amersham Bioscience, Piscataway, NJ, USA). The intensity of the bands was quantified using the Scion Image software (Scion, Frederick, MD, USA). Primary antibodies that detect proteins are listed in Table 2.

### 2.11. Statistical Analysis

Data represent mean ± SEM. Statistical analyses were performed using GraphPad Prism 5.01 (GraphPad Software Inc., La Jolla, CA, USA). The difference among the groups was analyzed using a one-way nonparametric ANOVA followed by Tukey’s multiple comparison test. Multiple comparison tests were only applied when a significant difference was determined using the ANOVA (*p <* 0.05).

## 3. Results

### 3.1. Effect of ML171 on Attenuation of Kidney Function and Histological Alteration in Kidney IRI 

**Figure 1:**
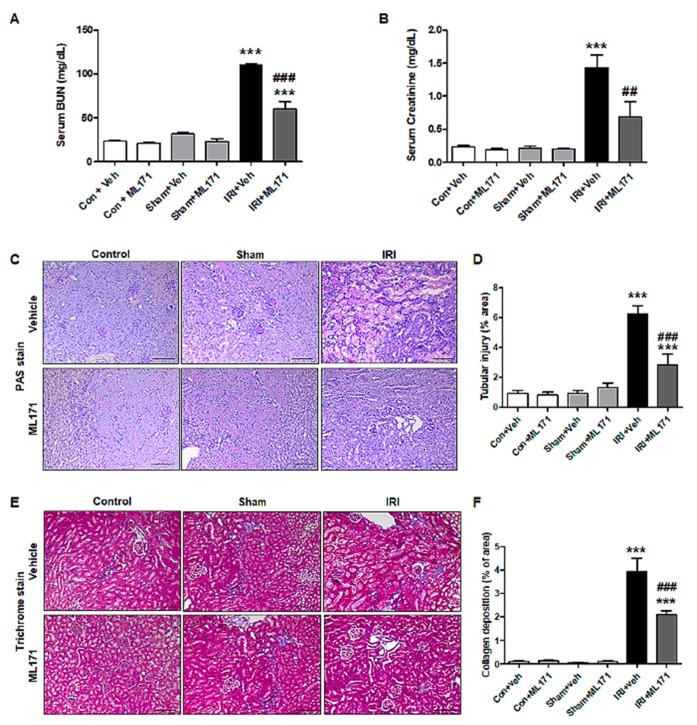
Effect of ML171 on attenuation of kidney function and histological alteration in renal ischemia-reperfusion injury (IRI). The levels of serum blood urea nitrogen (BUN) and creatinine were significantly reduced in ML171-pretreated IRI model (**A**,**B**). The periodic acid-Schiff (PAS)- and trichrome-stained kidney sections in IRI models showed that ML171 decreased tissue damage in renal tubular epithelial cells and collagen deposition (**C**–**F**). Data represent mean ± SEM. *** *p <* 0.001 vs. Con + Veh, ^##^ *p <* 0.01, ^###^ *p <* 0.001 vs. IRI + Veh. The difference among the groups was analyzed using a one-way nonparametric ANOVA followed by Tukey’s multiple comparison test.

Figure 1A,B shows the results of kidney function after IRI with treatment of ML171. BUN and Cr levels were significantly increased by IRI compared to the control groups. The levels of serum BUN and Cr were decreased in IRI mouse pretreated with ML171. The PAS and trichrome staining evaluated the histological changes in IRI models (Figure 1C–F). The histological analyses revealed that ML171 attenuated tubular necrosis, loss of the brush border, and cast formation in IRI kidney. Treatment with ML171 decreased collagen deposition in the IRI model.

### 3.2. Effect of ML171 on the Expression of NOX Family Subunits and Oxidative Stress Markers in IRI 

**Figure 2:**
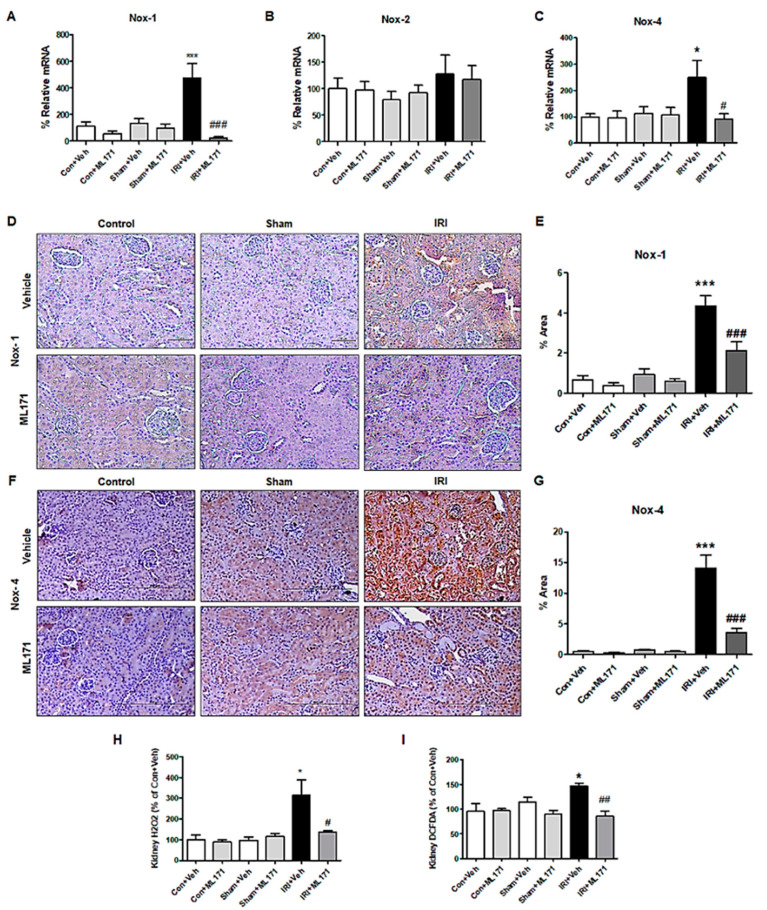
Effect of ML171 on nicotinamide adenine dinucleotide phosphate (NADPH) oxidase (NOX) family subunits and oxidative stress markers in IRI. The increased mRNA expression of NOX1 and NOX4 in IRI were significantly mitigated by ML171 (**A**–**C**). The immunohistochemical staining of NOX1 and NOX4 showed that the increased expression of NOX1 and NOX4 in IRI was decreased by ML171 (**D**–**G**). The increased reactive oxygen species (ROS) including H_2_O_2_ in the IRI model were significantly decreased by ML171 (**H**,**I**). Data represent mean ± SEM. * *p <* 0.05, *** *p <* 0.001 vs. Con + Veh, ^#^ *p <* 0.05, ^##^ *p <* 0.01, ^###^ *p <* 0.001 vs. IRI + Veh. The difference among the groups was analyzed using a one-way nonparametric ANOVA followed by Tukey’s multiple comparison test.

To investigate the changes in oxidative stress after IRI, the expression of NOX subunits and generation of ROS were evaluated. Among the NOX subunits, NOX1 and NOX4, but not NOX2 mRNA expression increased after IRI, which was significantly reduced by ML171 (Figure 2A–C). Immunohistochemical staining of NOX1 and NOX4 revealed an increased amount of NOX1 and NOX4 expression in the IRI model and a significant decrease by ML171 treatment (Figure 2D–G). ML171 attenuated the increase of intracellular H_2_O_2_ in the IRI kidney model (Figure 2H–I).

### 3.3. Effect of ML171 on Apoptosis in Kidney Tubular Cells Following IRI

**Figure 3:**
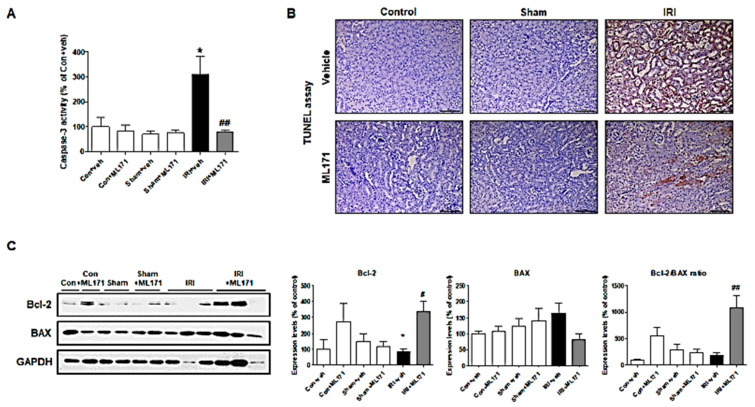
Effect of ML171 on apoptosis in kidney tubular cells following IRI. Caspase-3 activity was significantly decreased in IRI model after ML171 treatment (**A**). TUNEL assay showed that ML171 attenuated the apoptosis of kidney tubule cells (**B**). The Bcl-2 level and Bcl-2/Bax ratio were significantly increased in the IRI model after ML171 treatment (**C**). Data represent mean ± SEM. * *p <* 0.05 vs. Con + Veh, ^#^ *p <* 0.05, ^##^ *p <* 0.01 vs. IRI + Veh. The difference among the groups was analyzed using a one-way nonparametric ANOVA followed by Tukey’s multiple comparison test.

Caspase-3 activity decreased in the kidneys after ML171 treatment (Figure 3A), and TUNEL assay showed that apoptosis of kidney tubule cells decreased after MA171 treatment (Figure 3B). Figure 3C also showed that ML171 caused a significantly increased Bcl-2 level and Bcl-2/Bax ratio in the IRI model, suggesting the mitigating effect of ML171 on apoptosis.

### 3.4. Changes in Phosphorylated Proteins of MAPK Signaling Pathways in Kidney Tissues

**Figure 4:**
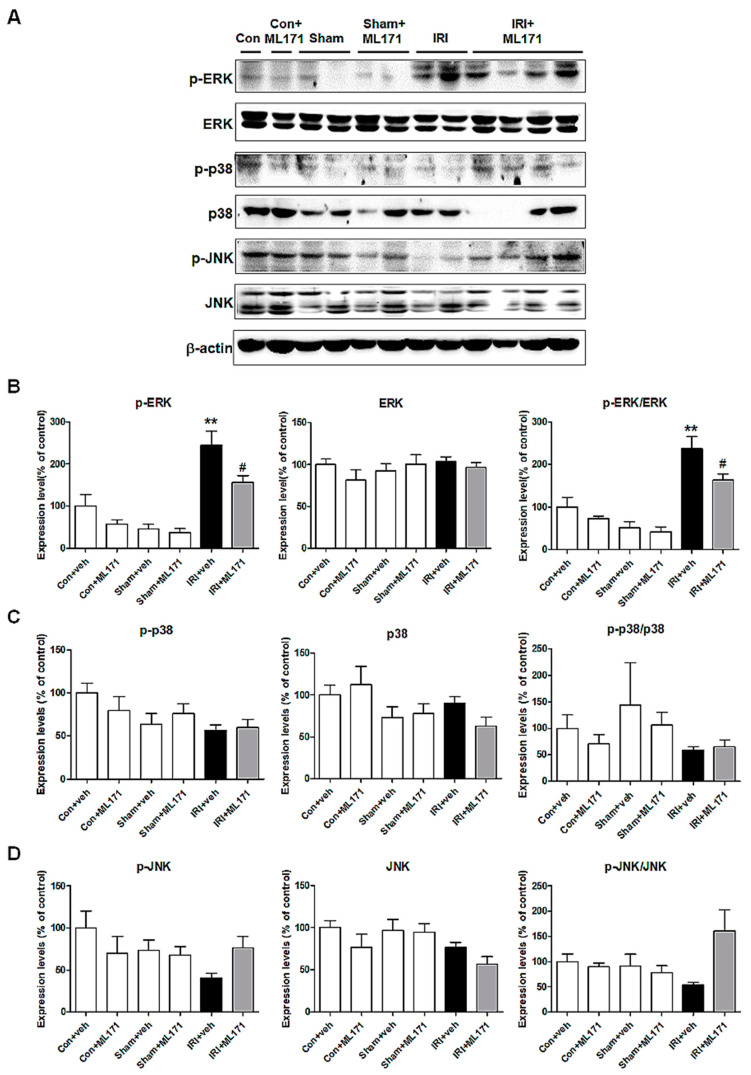
Changes in phosphorylated proteins of mitogen-activated protein kinase (MAPK) signaling pathways in kidney tissues. The genes of MAPK pathway determined the mechanism of oxidative stress-induced IRI. ML171 effectively attenuated increased p-ERK in the IRI model (**A**,**B**). The expression of phosphorylated p38 and JNK was not increased in the IRI model nor affected by ML171 (**C**,**D**). Data represent mean ± SEM. ** *p <* 0.01 vs. Con + Veh, # *p <* 0.05 vs. IRI + Veh. The difference among the groups was analyzed using a one-way nonparametric ANOVA followed by Tukey’s multiple comparison test.

We identified MAPK pathway genes to determine the oxidative stress mechanism induced by IRI. The Western blot of MAPK genes revealed that the phosphorylated extracellular signal-regulated kinase (p-ERK) was significantly increased in the IRI model, which was effectively attenuated by ML171. However, no difference in the expression of phosphorylated p38 and JNK was noted (Figure 4).

### 3.5. Effect of ML171 on NOX Subunit Expression and ROS Generation in H_2_O_2_-Treated MDCK Cells

**Figure 5:**
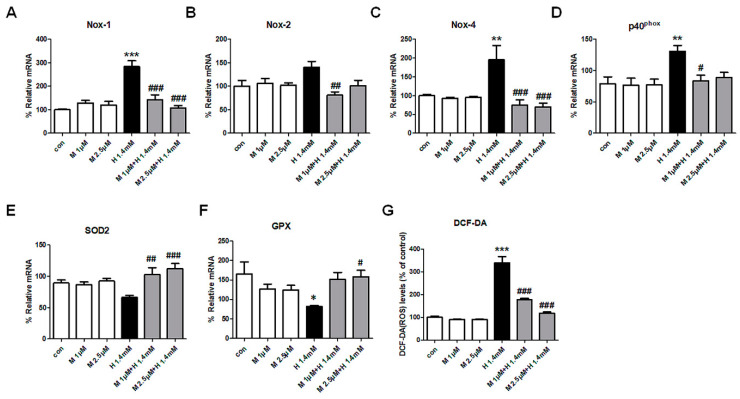
Effect of ML171 on H_2_O_2_-induced NOX subunit expression and ROS generation in MDCK cells. ML171 decreased NOX1, NOX4, and p40^phox^ expression in H_2_O_2_-treated MDCK cells (**A**–**D**). Oxidative stress-related enzymes of SOD2 and GPX production were mitigated by ML171 treatment (**E**–**G**). Data represent mean ± SEM. * *p <* 0.05, ** *p <* 0.01, *** *p <* 0.001 vs. Con, ^#^ *p <* 0.05, ^##^ *p <* 0.01, ^###^ *p <* 0.001 vs. H 1.4 mM. The difference among the groups was analyzed using a one-way nonparametric ANOVA followed by Tukey’s multiple comparison test.

MDCK cells were treated with H_2_O_2_ to induce NOX subunit expression and ROS generation. H_2_O_2_ increased expression of NOX1, NOX4, and p40^phox^, but not NOX2. ML171 attenuated the increased expression of NOX subunits induced by H_2_O_2_ in MDCK cells. H_2_O_2_ evoked a change in oxidative stress-related enzymes of SOD2 and GPX production, which was mitigated by ML171 treatment (Figure 5).

### 3.6. Effect of siRNA against NOX1 on H_2_O_2_-Treated MDCK Cells

**Figure 6:**
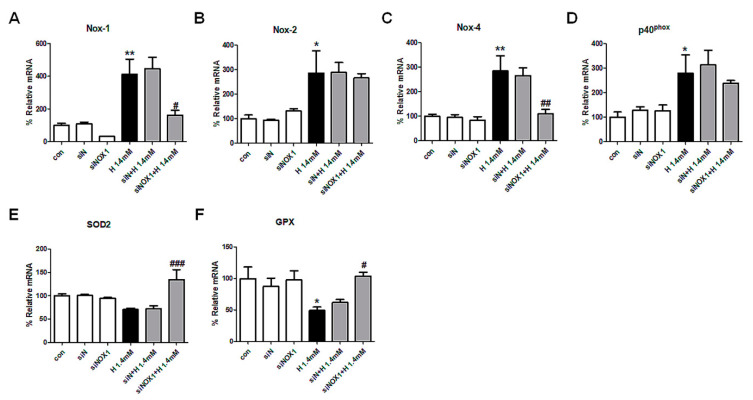
Effect of siRNA against NOX1 on H_2_O_2_-induced NOX subunit expression and ROS generation in MDCK cells. siRNA against NOX1 reduced NOX1 and NOX4 expression, however, did not affect NOX2 and p40^phox^ expression (**A**–**D**). Oxidative stress-related enzymes of SOD2 and GPX production were upregulated by siRNA against NOX1 treatment (**E**,**F**). Data represent mean ± SEM. * *p <* 0.05, ** *p <* 0.01 vs. Con, # *p <* 0.05, ^##^ *p <* 0.01, ^###^ *p <* 0.001 vs. H 1.4 mM. The difference among the groups was analyzed using a one-way nonparametric ANOVA followed by Tukey’s multiple comparison test.

MDCK cells were transfected with siRNA against NOX1 to evaluate whether the protective effect of ML171 was mediated with NOX1 and the expression of NOX4 was related with that of NOX1 in MDCK cells. Compared to nontargeting siRNA, siRNA against NOX1 showed a decreased NOX1 expression after H_2_O_2_ induction. Transfection with siRNA against NOX1 also upregulated SOD2 and GPX mRNA, which is consistent with the effect of ML171 treatment. Surprisingly, H_2_O_2_-induced expression of NOX4 mRNA was inhibited in MDCK cells with siRNA against NOX1, whereas NOX2 and p40^phox^ expression remained increased (Figure 6).

### 3.7. Effect of ML171 on the Apoptosis Induced by H_2_O_2_ in MDCK Cells

**Figure 7:**
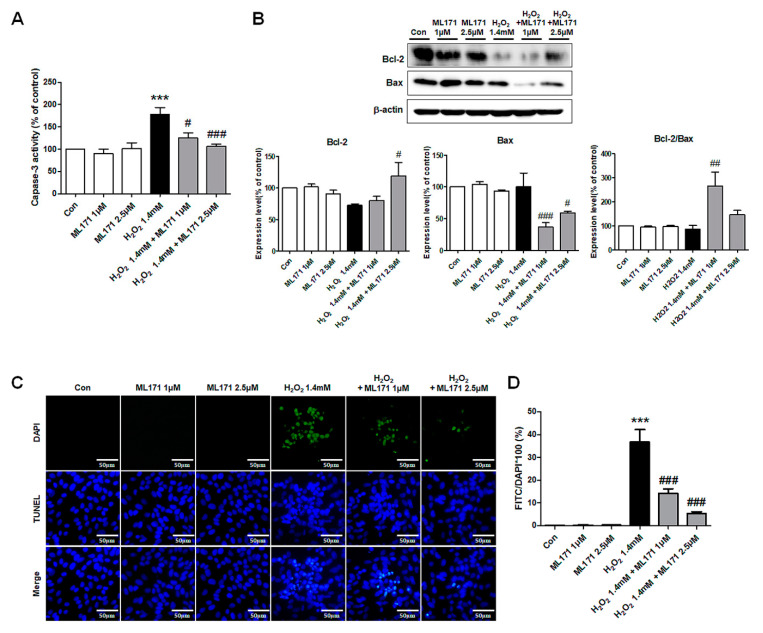
Effect of ML171 on the apoptosis induced by H_2_O_2_ in MDCK cells. Caspase-3 activity was significantly increased after H_2_O_2_ induced oxidative stress in MDCK cells and was significantly decreased by ML171 treatment (**A**). ML171 attenuated the changes in the expression of Bax and the Bcl-2/Bax ratio in H_2_O_2_-treated MDCK cells (**B**). TUNEL assay showed that H_2_O_2_-induced apoptosis was significantly reduced by ML171 treatment (**C**,**D**). Data represent mean ± SEM. *** *p <* 0.001 vs. Con, ^#^ *p <* 0.05, ^##^ *p <* 0.01, ^###^ *p <* 0.001 vs. H 1.4 mM. The difference among the groups was analyzed using a one-way nonparametric ANOVA followed by Tukey’s multiple comparison test.

The effect of ML171 on the apoptosis induced by oxidative stress was assessed in MDCK cells. Caspase-3 activity increased significantly after H_2_O_2_ treatment and decreased after ML171 treatment in MDCK cells (Figure 7A). ML171 also attenuated the changes in the expression of Bax and Bcl-2/Bax ratio in H_2_O_2_-treated MDCK cells (Figure 7B). The TUNEL assay showed that ML171 decreased the H_2_O_2_-induced apoptosis in tubule cells (Figure 7C,D).

### 3.8. Changes in Phosphorylated Proteins of MAPK Signaling Pathways in MDCK Cells

**Figure 8:**
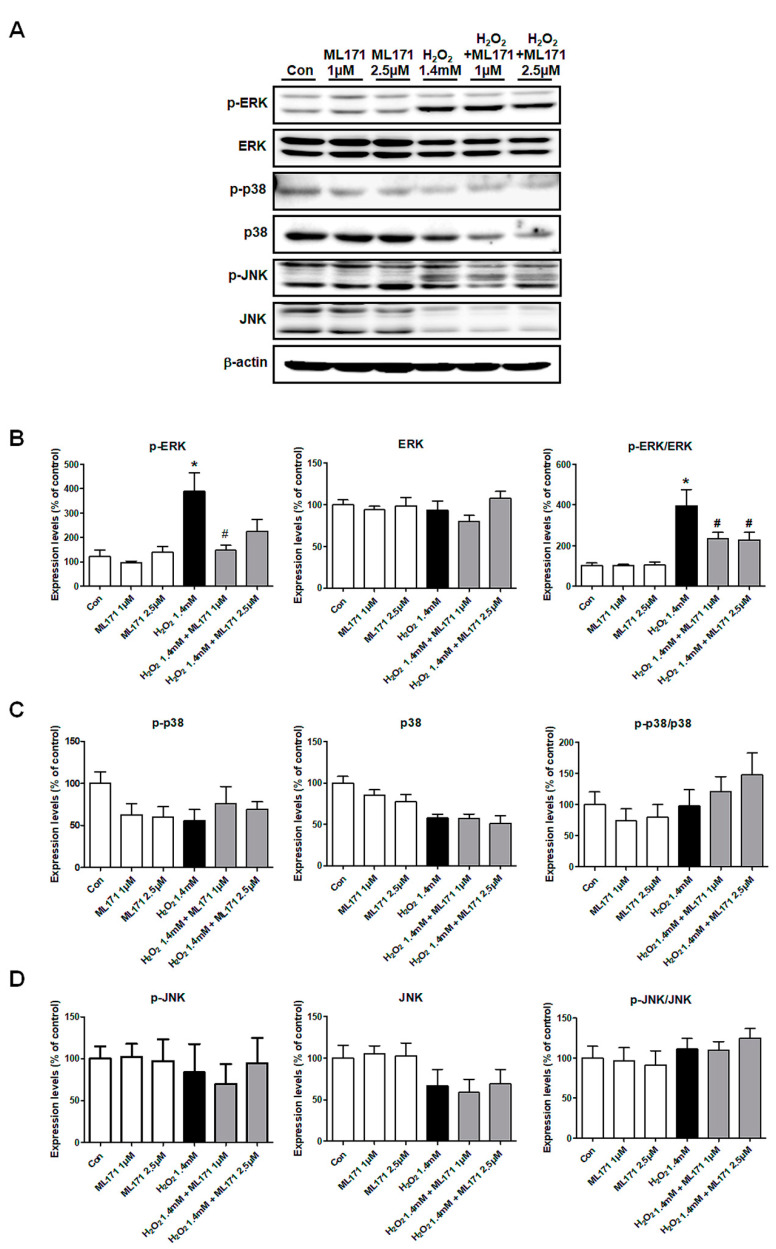
Changes in phosphorylated proteins of MAPK signaling pathways in MDCK cells. Consistent with the results from the in vivo experiment, the increased p-ERK and p-ERK/ERK ratio after H_2_O_2_ treatment was significantly decreased after ML171 treatment in MDCK cells (**A**,**B**). There were no significant differences in the expression of phosphorylated p38 and JNK after ML171 treatment (**C**,**D**). Data represent mean ± SEM. * *p <* 0.05 vs. Con, ^#^ *p <* 0.05 vs. H 1.4 mM. The difference among the groups was analyzed using a one-way nonparametric ANOVA followed by Tukey’s multiple comparison test.

We investigated the mechanism with which ML171 blocked oxidative stress and apoptosis in MDCK cells. The p-ERK and p-ERK/ERK ratio significantly increased after H_2_O_2_ treatment and was significantly reduced by ML171 in MDCK cells. p38 and JNK expressions were not affected by H_2_O_2_ and ML171, which was consistent with the in vivo experiment results (Figure 8).

## 4. Discussion

The present study demonstrated that NOX1 inhibition by ML171 attenuated kidney IRI in the mouse model. The ischemic injury decreased significantly in the ML171-treated IRI group compared to that in the IRI group. The tissue injury was associated with the increased ROS production and NOX expression, which was reversed by ML171 treatment. H_2_O_2_ evoked changes in oxidative stress-related enzymes of SOD2 and GPX production in MDCK cells, which was mitigated by NOX1 inhibition with ML171 and siRNA against NOX1. Treatment with ML171 and transfection with siRNA against NOX1 decreased the expression of NOX1 induced by H_2_O_2_ exposure in MDCK cells. ML171 caused a significant increase in the Bcl-2 level and decrease in caspase-3 activity in IRI mice and H_2_O_2_-treated MDCK cells. ML171 affected ERK signaling by the phosphorylation of ERK in kidney tissues and tubular cells. The present study was the first to suggest that NOX1 inhibition could protect kidney IRI by inhibition of ROS-mediated ERK signaling.

Ischemic damage of the kidney causes acute kidney injury, which is responsible for the hypoxic damage induced by the production of ROS as well as the decrease in kidney blood flow [22,23]. ROS acts as signaling molecules including regulation of vascular tone, monitoring of oxygen tension, and signal transduction from membrane receptors in physiological situations, but in excess, it causes tissue damage [24,25,26]. NOX, one of the main sources of ROS, catalyzes the transfer of electrons from NADPH to molecular oxygen to produce ROS [27].

NOX4 is the most distributed NOX isoform in the kidney and has been studied in various kidney diseases. NOX4 expression in proximal tubular cells increased after exposure to high glucose, and NOX4 inhibition with GKT136901 decreased albuminuria in diabetic mice [28,29]. NOX4 deficiency was associated with the increased tubular injury after IRI [30], and hypoxia to kidney tubule cell upregulated NOX4 expression via a TGF-β1/Smad signaling pathway [31]. NOX2 also plays a role especially in the development of diabetic nephropathy, and its expression was upregulated in the kidneys of diabetic mice [32,33,34]. NOX2 is the classic phagocytic NOX and its main role is free radical generation [35,36]. It has been reported that NOX2 inhibition could prevent kidney damage and delayed graft function after IRI by the inhibition fibrosis and oxidative stress [37].

NOX1 is also expressed in the kidney cortex [38,39,40], and the associations with ischemic injury have been reported in other organs. NOX1 was a therapeutic target in ischemic retinopathy and IRI in the heart [41,42]. The role of NOX1 in kidney injury has been reported in cisplatin nephrotoxicity. Cardamonin, a flavone with anti-inflammatory activity, inhibited NOX1 expression in the cisplatin nephrotoxicity model, decreasing inflammation and apoptosis in the injured kidney [13]. The study on a NOX inhibitor has also revealed its protective effect in the kidney IRI. Apocynin, a nonspecific NOX inhibitor, ameliorated the histological damages after IRI by reducing oxidative stress markers, demonstrating that NOX1 was associated with kidney injury and downregulation of NOX could prevent kidney damage. However, it was difficult to attribute the action specifically to NOX1 inhibition, since two drugs did not selectively inhibit NOX1. In contrast, ML171 is a known potent NOX1 inhibitor with isoform selectivity only for NOX1 [21]. In the present study, NOX1 expression significantly increased in the kidney after IRI and MDCK cells treated with H_2_O_2_. ML171 effectively suppressed NOX1 upregulation induced by IRI, and the suppression of NOX1 by ML171 was also observed in H_2_O_2_-treated tubular cells, which are the main site of IRI. The treatment of ML171 showed improvement of serum BUN and Cr levels in the IRI group, suggesting significant amelioration of the ischemic injury. Therefore, this is the first study to demonstrate the renoprotective effect of NOX1 selective inhibition through the IRI animal model.

The expression of NOX1, NOX2, and NOX4 is closely related to each other [43]. Angiotensin II increases oxidative stress by upregulating the kidney cortical gene product for NOX1 and p22^phox^, a catalytic core of the NOX [38]. Rats with the silenced p22^phox^ gene reduced NOX1, NOX2, and NOX4 expression in the kidney cortex during infusion of angiotensin II [40]. Aoyama et al. showed that angiotensin II-induced NOX4 expression was inhibited by NOX1 knock-out hepatocytes, suggesting that NOX1 could induce NOX4 upregulation [44]. Our results are consistent with the previous study in that NOX4 expression was downregulated after transfection with siRNA against NOX1 into H_2_O_2_-treated MDCK cells. The reduced expression of NOX4 by ML171 treatment might be associated with the causal interaction between NOX1 and NOX4, which could also be explained by the downregulation after reduced oxidative stress by NOX1 inhibition or a partial inhibitory effect of ML171 on NOX4 [45].

H_2_O_2_ is a major marker of oxidative stress, giving rise to tissue injury after IRI [46,47]. In the present study, H_2_O_2_ increased significantly in the kidney after IRI. DCFDA, a compound used to measure generalized oxidative stress including intracellular H_2_O_2_ production, also significantly increased in the tubular cells treated with H_2_O_2_. Therefore, the injury with H_2_O_2_ in the MDCK cells is comparable to the injury induced by IRI in the kidney. SOD2 and GPX are two important components of the defensive mechanisms against oxidative stress by preventing the reactive free radical formation [48,49,50,51]. ML171 reduced H_2_O_2_ in the IRI mouse, and the effect was manifested as a subsequent increase in SOD2 and GPX in MDCK cells, suggesting the prevention of kidney damage with oxidative stress by selective NOX1 inhibition.

MAPK is associated with the activation of NOX1 [52]. ERK and p38 as well as JNK are members of MAPK that have proline-directed kinase activity. However, a certain stimulus selectively activates a specific member of the MAPK pathway [53]. Among them, ERK signaling pathway was involved in the present study. Only phosphorylation of ERK increased after ischemia and H_2_O_2_ exposure. ERK phosphorylates p47^phox^ and contributes to NOX1 complex assembly [53]. Our results are consistent with the previous report that suggested the association of ERK with the cellular survival of the kidney with IRI [54].

We also investigated the possible mechanisms by which NOX1 inhibition prevented tubular injury. ROS generated by ischemia or oxidative stress are well-known inducers of apoptosis by activating caspase-3 in kidney tubular cells [6]. It was supported by TUNEL-positive kidney tubular epithelial cells and decreased Bcl-2/Bax ratios [55,56]. ML171 treatment before IRI significantly decreased caspase-3 activity, decreased the number of TUNEL-positive cells, and increased Bcl-2/Bax ratios compared with the IRI group. This suggests that the renoprotective effects of NOX1 inhibition are mediated via modulating kidney tubular cell apoptosis after IRI.

Our study has several limitations. First, our results were not validated with the other oxidative stress injury models such as cisplatin nephrotoxicity or contrast-induced nephropathy. However, the IRI model is a prototype of oxidative stress injury to kidney, and, although other models may show slight differences, we tried to determine NOX1′s role in the basic injury model. Second, further experiments with a more specific way to inhibit NOX1 expression are needed to find NOX1′s effect in IRI. GKT771, a highly selective NOX1 inhibitor [57] or NOXA1ds, a peptide NOX1 inhibitor with greater specificity and isoform selectivity [58], might reveal the specific effect of NOX1-selective inhibition. Third, our experiment was not performed on murine or human kidney epithelial cell lines. An animal toxicity test and further experiment with human kidney epithelial cells must be accompanied to apply the present results to human medicine. Nevertheless, this is the first study to investigate the renoprotective effect of NOX1 inhibition on the kidneys and kidney tubule cells, which makes it a candidate for treatment of acute kidney injury.

## 5. Conclusions

NOX1-selective inhibition by ML171 attenuated kidney IRI via inhibition of ROS-mediated ERK signaling. NOX1 inhibition by ML171 and siRNA against NOX1 reduced the oxidative stress-induced apoptosis in MDCK cells, indicating NOX1 selective inhibition’s potential as a therapeutic target for acute kidney injury associated with ROS generation and subsequent apoptosis.

## 6. Patent

The results of this paper were patented under the name “Composition for preventing or treating ischemia-reperfusion injury comprising NADPH oxidase 1” on 20 April 2020 (Number: 10-2020-0047663).

## Figures and Tables

**Table 1:** Oligonucleotide primer sequences.

Gene	Forward Primer (5′–3′)	Reverse Primer (5′–3′)
Mouse Nox-1	CAG GCC ATG GAT GGA TCT CT	ATG TTT GGA GAC TGG ATG GGA TT
Mouse Nox-2	GCT CTC TCT GAC ATC GGT GAC A	CGA GTC ACG GCC ACA TAC AG
Mouse Nox-4	CAC CAA ACA CAG AAG CAC AAG AC	AAA GCA GGG TAT CAC TCC ATG AA
Mouse GAPDH	TAA AGG GCA TCC TGG GCT ACA CT	TTA CTC CTT GGA GGC CAT GTA GG
Dog Nox-1	CCC CGC TGA GTC TTG GAA	TAA AAT CGG AGA ATC CTT TCA AGA A
Dog Nox-2	GAC ACG CAC GCC TTT GAG T	CCT GCA TCT GGG TCT CTA GCA
Dog Nox-4	CAC TCT TCG GAC TAT ACT GCA TGA TC	TCA TCC CCT GAG CCA AGA AT
Dog p40phox	GGG AAG ACA TCG CCC TGA AT	ACA GCA GCC GCA CCA GAT
Dog SOD2	CGC CGC CTA CGT GAA CA	CTC CAG CGC CTC CAG ATA CT
Dog GPX	GAA TGT GGC GTC GCT CTG A	CGC TGC AGC TCG TTC ATC T
Dog GAPDH	GAT GCC CCC ATG TTT GTG A	TTT GGC TAG AGG AGC CAA GCA

**Table 2:** List of antibodies used in immunohistochemistry and immunoblotting.

Antibodies	Cat. No.	Company
Dog Bcl-2	ab117115	Abcam
Dog Bax	2772S	Cell signaling
Dog β-actin	A1978	Sigma
Dog p-Erk1 (pT202/pY204) + p-Erk2(pT185/pY187)	ab4819	Abcam
Dog Erk1+Erk2	ab17942	Abcam
Dog p-p38 (phospho T180+Y182)	ab4822	Abcam
Dog p38 (M138)	ab31828	Abcam
Dog p-JNK1 + p-JNK2 (phospho T183+Y185)	ab4821	Abcam
Dog JNK1 + JNK2 + JNK3	ab179461-1	Abcam
Mouse Bcl-2	2876	Cell signaling
Mouse Bax	2772	Cell signaling
Dog, mouse NOX-1	Ab121009	Abcam
Mouse NOX-4	MA5-32090	Invitroten
Mouse a-SMA	Ab5694	Abcam
Mouse fibronectin	Ab2413	Abcam
Mouse GAPDH	2118S	Cell signaling
Mouse p-AKT (Ser473)	9271	Cell signaling
Mouse AKT	9272	Cell signaling
Mouse p-ERK (Thr202/Tyr204)	4370S	Cell signaling
Mouse ERK	9102	Cell signaling
Mouse p-JNK (Thr183/Tyr185)	9251S	Cell signaling
Mouse JNK	9252S	Cell signaling
Mouse p-p38 (Thr180/Tyr182)	9211S	Cell signaling
Mouse p38	9212S	Cell signaling

## Abbreviations

BUN	Blood urea nitrogen
Cr	Creatinine
IRI	Ischemia-reperfusion injury
KHIDI	Korea Health Industry Development Institute
MAPK	Mitogen-activated protein kinase
MDCK	Madin–Darby Canine Kidney
NADPH	Nicotinamide adenine dinucleotide phosphate
PAS	Periodic acid-Schiff
ROS	Reactive oxygen species
TdT	Terminal deoxynucleotidyl transferase
